# Evaluation of the Characteristics and Coating Film Structure of Polymer/Ceramic Pressure-Sensitive Paint

**DOI:** 10.3390/s18114041

**Published:** 2018-11-20

**Authors:** Yosuke Sugioka, Kazuto Arakida, Miku Kasai, Taku Nonomura, Keisuke Asai, Yasuhiro Egami, Kazuyuki Nakakita

**Affiliations:** 1 Department of Aerospace Engineering, Graduate School of Engineering, Tohoku University, 6-6-01 Aramakiaza-Aoba, Aoba-ku, Sendai, Miyagi 9808579, Japan; arakida.kazuto@aero.mech.tohoku.ac.jp (K.A.); kasai.miku@aero.mech.tohoku.ac.jp (M.K.); nonomura@aero.mech.tohoku.ac.jp (T.N.); asai@aero.mech.tohoku.ac.jp (K.A.); 2 Department of Mechanical Engineering, Aichi Institute of Technology, 1247 Yachigusa, Yakusa-cho, Toyota, Aichi 4700392, Japan; egami@aitech.ac.jp; 3 Aeronautical Technology Directorate, Japan Aerospace Exploration Agency, 7-44-1 Jindaiji-Higashi, Chohu, Tokyo 1828522, Japan; nakakita@chofu.jaxa.jp

**Keywords:** pressure-sensitive paint (PSP), polymer/ceramic PSP (PC-PSP), unsteady measurement

## Abstract

Polymer/ceramic pressure-sensitive paint (PC-PSP), which incorporates a high percentage of particles in the binder layer, is proposed in order to improve the characteristics of PSP. The procedure for embedding particles into the binder layer was modified. In the conventional procedure, dye is adsorbed onto a polymer/ceramic coating film (denoted herein as a dye-adsorbed (D-adsorbed) PSP). In the new procedure, the mixture of a dye and particles is adsorbed onto a polymer coating film (denoted herein as the particle/dye-adsorbed (PD-adsorbed) PSP). The effect of particle mass content on PSP characteristics was investigated. In addition, the effect of solvent on PSP characteristics and film structure were evaluated for the PD-adsorbed PSP. As a result, the difference in the PSP characteristics between the two types of PSP was clarified. Although surface roughness and time response increase with increased mass content of particles for both D- and PD-adsorbed PSPs, the critical pigment volume concentration (CPVC) for the PD-adsorbed PSP is smaller than that of the D-adsorbed PSP (88 wt% and 93 wt%, respectively). The PD-adsorbed PSP has a higher frequency response comparing with the D-adsorbed PSP while maintaining the same surface roughness. Observation by scanning electron microscope showed that the CPVC of the PC-PSP is governed primarily by surface structure. The coating film structure can be roughly classified into two states depending on the particle mass content. One is a state in which the coating film consisted of two layers: a lower particle-rich layer and an upper polymer-rich layer. This type of structure was observed in the PD-adsorbed PSP as well as in the D-adsorbed PSP. In the other state, polymer and particles are homogeneously distributed in the film, and pores are formed. This difference in the coating structure results in a change in the time response.

## 1. Introduction

Pressure-sensitive paint (PSP) has attracted a great deal of attention from the aerospace community as a new method by which to measure pressure fields at high spatial resolution [1]. PSP is an optical pressure-measurement technique based on oxygen quenching of luminescence. Typically PSP coatings comprise dye molecules (luminophores) and an oxygen-permeable binder. Since the intensity of the light emitted from the PSP layer depends on the partial pressure of oxygen, the air pressure over the PSP coating can be calculated from the measured luminescence intensity.

PSP is used to measure time-resolved (unsteady) pressure fields as well as time-averaged (steady) pressure fields [2,3]. Fast-responding PSP with a highly-diffusive porous binder must be used in order to measure unsteady pressure fields because the time response of PSP is governed by the diffusion process through the binder. Three major types of materials have been used as a porous binder for fast-responding PSPs: thin-layer chromatography (TLC) plates [4], anodized aluminum (AA) [5,6], and polymer/ceramic (PC) composites [7,8,9,10,11].

TLC-PSP and AA-PSP have been widely used for unsteady pressure measurement, but the shape and the material of test articles to which these types of PSP have been applied are limited. On the other hand, PC-PSP can be applied to any test article by a spraying procedure.

PC-PSP is composed of a polymer matrix and small ceramic particles. Previous studies showed that the materials and their compounding ratios affect characteristics such as luminescent intensity, pressure and temperature sensitivity, and response time [7,8,9,10,11]. Ponomarev et al. [9] investigated the effect of particle content on the response times of several kinds of PC-PSP. This study first revealed that the response time of PSP was drastically improved if the volume concentration of the particles additive was above a critical value, i.e., the critical pigment volume concentration (CPVC). Scroggin et al. [7] and Gregory et al. [8] also studied the effect of materials and their mixing ratios on the PSP characteristics. Their study led to the development of a high-performance PC-PSP, which is commercially available from Innovative Scientific Solutions Inc. (Dayton, OH, USA) and is used in numerous wind tunnel tests. Sakaue et al. [10] showed that the signal level of PC-PSP with bathophen ruthenium could be maximized by controlling the ratio of the polymer content. Kitashima et al. [11] studied the characteristics of PC-PSP composed of a fluoric polymer, titanium dioxide (TiO_2_) particles, and PtTFPP. The effects of particle size, particle content, and dye application method on the PC-PSP characteristics were investigated systematically. Their research showed that the response time decreased drastically when the particle content was greater than 85 wt%, accompanied by the change in the coating structure. Furthermore, it was found that the dye application method also affected the time response of the PC-PSP.

In recent years, there has been a great demand for the application of PC-PSP to industrial wind tunnel tests. However, the effect of surface roughness of PC-PSP on flow cannot be negligible in such tests. PC-PSP contains a large amount of particles in the binder in order to increase the gas diffusivity, but these particles also make the painted model surface rougher than a clean model. Sugioka et al. [12] reported that the shock-wave location on an airfoil was highly sensitive to the surface roughness of PSP in a transonic wind tunnel test, as expected.

In order to reduce the surface roughness, Sugioka et al. [12] modified the formulation of a polymer/ceramic binder. As a result, they developed a PC-PSP that has an arithmetic surface roughness of 0.5 μm and a cutoff frequency of 3 kHz. This PSP was applied to transonic buffet studies of a two-dimensional airfoil [12] and a three-dimensional wing [13], and the capability of the PC-PSP was confirmed.

**Figure 1:**
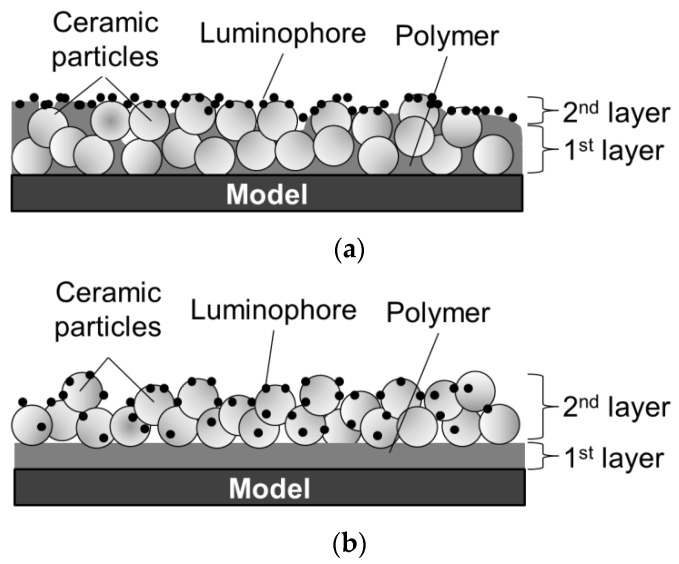
Schematic diagrams of PC-PSPs suggested in previous studies. (**a**) Dye-adsorbed (D-absorbed) PSP; (**b**) particle/dye-absorbed (PD-absorbed) PSP.

Numerous studies have examined the improvement of PC-PSP characteristics. For instance, Sato et al. [14] proposed a new coating procedure to improve the time response of PC-PSP. For conventional PC-PSPs, ceramic particles are added to a polymer solution, which is sprayed together on a test article, as shown in Figure 1a. On the other hand, in Sato’s procedure, ceramic particles are added to a dye solution and are adsorbed onto a pre-coated polymer film. They reported that a faster time response could be achieved using the new coating procedure. Moreover, the surface roughness of Sato’s PC-PSP is lower than the admissible roughness in a conventional wind tunnel test. It has been estimated that the coating film structure is as shown in Figure 1b, but micro-scale observation of the coating film structure has not been conducted, and structure is not well understood. In the present paper, the conventional PC-PSP and the new PC-PSP are referred to, respectively, as the dye-adsorbed (D-adsorbed) PSP and the particle/dye adsorbed (PD-adsorbed) PSP.

The goals of the present paper are to improve the characteristics of PC-PSP by using a new coating procedure and to compare the characteristics of the new and conventional coating methods. The effect of particle mass content on PSP characteristics such as pressure and temperature sensitivities, surface roughness, and frequency response were investigated for both methods. In addition, the effect of solvent for a dye/particle solution was evaluated. Finally, the mechanisms of the changes in the characteristics are discussed according to microstructures imaged by a scanning electron microscope (SEM).

## 2. Materials and Methods

The materials of the PC-PSP are the same as those used in [12]. The polymer/ceramic binder consisted of a commercially available ester polymer, titanium-dioxide (TiO_2_) particles with a diameter of 250 nm (rutile type, Teyka, Osaka, Japan). We employed TiO_2_ because it has high whiteness, appropriate particle size, high dispersibility, and high chemical stability. The oxygen sensing dye used in the present study is platinum tetra (pentafluorophenyl) porphyrin (PtTFPP) (PtT975, Frontier Scientific, Logan, UT, USA). Polymer solutions were prepared for the PD- and D-adsorbed PSPs by dissolving 0.25 g of polymer in 10 mL and 15 mL of toluene (Wako Pure Chemical, Osaka, Japan), respectively. The amount of the solvent for the D-adsorbed PSP is larger than that for the PD-adsorbed PSP because the coating property of polymer/ceramic composite is worse than that of polymer solution. A dye solution was prepared by mixing 2 mg of PtTFPP and 10 mL of solvent. The solvent for the dye solution was toluene. In addition, for the PD-adsorbed PSP, a mixture of toluene and methanol was used as the solvent for the dye and TiO_2_ solution, because it was reported that the use of methanol improves the time response of PC-PSP [14]. The proportion of the methanol was 0, 20, 50, 80, and 100%.

As mentioned in the Introduction, sample coupons were prepared by two coating procedures. TiO_2_ particles are added to the polymer solution for the conventional D-adsorbed PSP, whereas TiO_2_ particles are added to the dye solution for the PD-adsorbed PSP. The solution containing TiO_2_ (i.e., polymer solution for the D-adsorbed PSP and dye solution for the PD-adsorbed PSP) is stirred for approximately 12 h.

In the present study, the mass content of TiO_2_ particles is varied between 80-96 wt% and its effects on the PSP characteristics are investigated. The particle mass content is defined as the weight ratio of TiO_2_ particles to the total weight of particles and polymer. Table 1 shows the preparation conditions of the PC-PSPs.

The painting procedure of PC-PSP is as follows: first, polymer or polymer/ceramic film was formed on aluminum plates using a spray gun. After drying the coating film for more than 12 h, a particle/dye or dye solution was sprayed onto the pre-coated film. Three types of sample coupons were used: 25-mm-square coupons for the roughness measurement, 15-mm-square coupons for the static calibration test, and 20-mm-square coupons with a hole for the frequency response testing.

**Figure 2:**
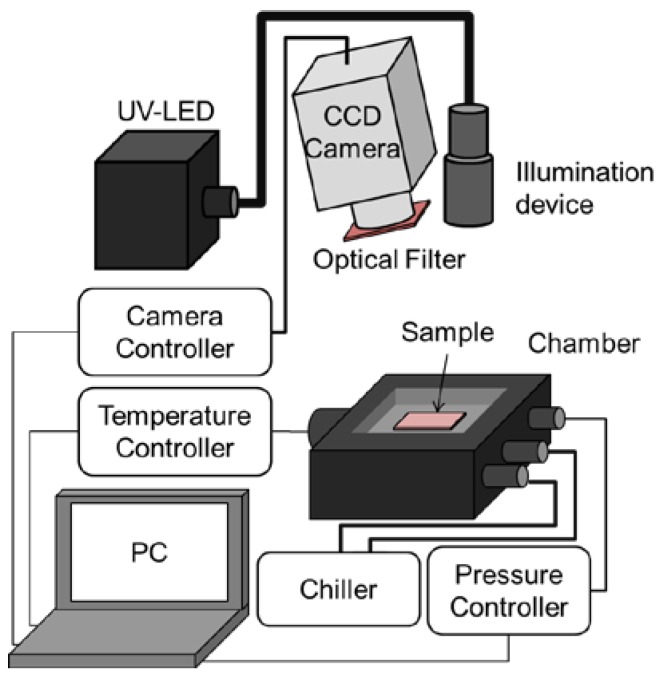
Static calibration system.

The pressure and temperature sensitivities of PSP were evaluated using a chamber in which the inner pressure and the base temperature could be controlled. The schematic diagram of the static calibration system is shown in Figure 2.

Sample coupons were illuminated with a UV-LED (IL-106, Hardsoft, Kraków, Poland), the center wavelength of which was 390 nm, and the emission from PSP was collected with a 16-bit CCD camera (ORCA II-BT1024, Hamamatsu Photonics, Hamamatsu, Japan). An optical filter (650 ± 20 nm, PB0650/040, Asahi Spectra, Tokyo, Japan) was mounted in front of the camera lens. The pressure *P* inside the chamber and the temperature *T* of the coupons were varied in the ranges of 5 to 140 kPa and 283 to 333 K, respectively. The luminescent intensity *I* under each condition was normalized by the intensity under the reference condition of *P*_ref_ = 100 kPa and *T*_ref_ = 293 K. The intensity ratios were fitted to Equations (1) and (2), and the gradient at the reference condition was determined as the pressure or temperature sensitivity:

(1) $$\frac{I_{\mathrm{ref}}}{I}=A_{1}\left(T_{\mathrm{ref}}\right)+A_{2}\left(T_{\mathrm{ref}}\right)\frac{P}{P_{\mathrm{ref}}}+A_{3}\left(T_{\mathrm{ref}}\right){\left(\frac{P}{P_{\mathrm{ref}}}\right)}^{2},$$

(2) $$\frac{I_{\mathrm{ref}}}{I}=C_{1}\left(P_{\mathrm{ref}}\right)+C_{2}\left(P_{\mathrm{ref}}\right)T+C_{3}\left(P_{\mathrm{ref}}\right)T^{2},$$

Frequency response tests were conducted using an acoustic resonance tube [15]. This device can generate pressure fluctuations on the order of kilopascals over the frequency range from 0.15 to 10 kHz. A PSP sample coupon was placed at the endcap and excited with a UV laser (RV-1000TH, Ricoh, Yokohama, Japan), the wavelength of which was 400 nm. The emission from the coupon was measured using a photomultiplier tube (PMT) (H5784-02, Hamamatsu Photonics, Hamamatsu, Japan). A band-pass optical filter (650±20 nm) was placed in front of the PMT. The PMT signal and a pressure transducer signal were acquired simultaneously with a data acquisition (DAQ) device (USB-6251, National Instruments, Austin, TX, USA). The recorded signals were then converted to pressure using the calibration equation of PSP obtained from the static calibration test. The pressure fluctuations were then phase averaged and fitted by a sine curve. By comparing the obtained amplitude and phase, the gain and phase shift were calculated for PSP data. The details of the data-acquisition procedure and the analysis methods are shown in a previous study [15]. The cut-off frequency is defined as the frequency at which the decay of the gain becomes −3 dB.

The surface roughness of PSP sample coupons was evaluated using a laser microscope (VK-X210 3D, KEYENCE, Osaka, Japan) and bundled software (VK-H1XV, KEYENCE). Images of nine points on a sample coupon were acquired. The arithmetic average roughness *Ra* at each point was then calculated and averaged.

The microstructure of PC-PSP was imaged with a field emission scanning electron microscope (FE-SEM) (JSM-6500F, JEOL, Tokyo, Japan). The surfaces of the sample coupons were coated with an approximately 10-nm-thick gold coating. For quantitative evaluations, the brightness of the SEM images was standardized so that the average was 0 and the standard deviation was 1. Skewness and kurtosis of the brightness histogram were calculated, and the surface structures were quantified.

## 3. Results

### 3.1. Effect of Coating Procedure and Particle Mass Content

**Figure 3:**
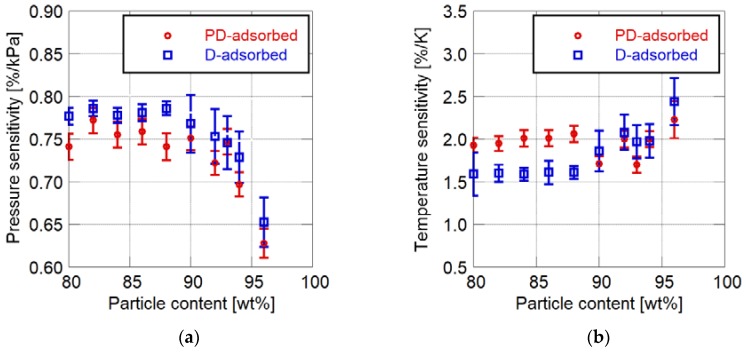
Static sensitivity characteristics related to particle mass content. (**a**) Pressure sensitivity; (**b**) Temperature sensitivity.

Figure 3 shows the changes in pressure and temperature sensitivities related to the particle mass content. Here, toluene is used as the solvent of the particle/dye solution for the PD-adsorbed PSP. The error bars in below figures show the standard deviations of the sensitivities in the analytical area on a sample coupon.

The addition of a large amount of particles affects the static characteristics. The pressure sensitivity of the PC-PSP decreases with the increase in the particle mass content. In particular, the pressure sensitivity rapidly becomes lower when the particle mass content exceeds 90 wt%. The temperature sensitivity of the D-adsorbed PSP increases when 90 wt% or more particles are added. On the other hand, the temperature sensitivity of the PD-adsorbed PSP gradually changes with the particle mass content.

Figure 3 indicates that the sensitivity characteristics of PC-PSP are also related to the coating procedure as well as the particle mass content. In most cases, the pressure sensitivity of the D-adsorbed PSP is larger than that of the PD-adsorbed PSP. For PSP, the particle mass content of which is less than 88 wt%, the temperature sensitivity of the D-adsorbed PSP is smaller than that of the PD-adsorbed PSP. From the above discussion, the D-adsorbed PSP has superior sensitivity characteristics to the PD-adsorbed PSP.

**Figure 4:**
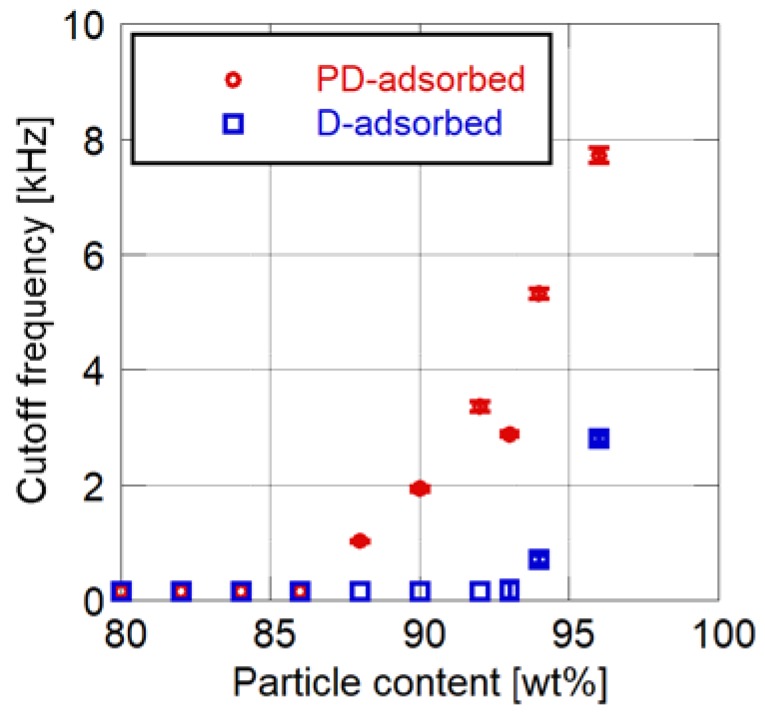
Cutoff frequency related to particle content.

**Figure 5:**
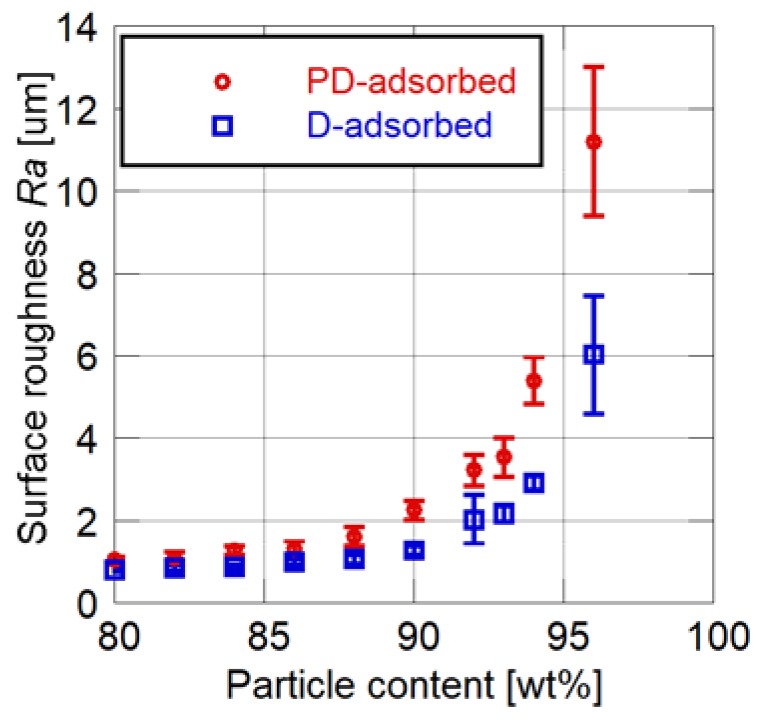
Surface roughness related to particle content.

Figure 4 and Figure 5 show the effects of particle mass content on the cutoff frequency and surface roughness of PSP, respectively. Toluene is used as the solvent of the particle/dye solution for the PD-adsorbed PSP. The error bars for the cutoff frequency show the standard deviation in four times of the measurement, and the error bars for the surface roughness show the standard deviation of the *Ra* measured at nine arbitrary points.

Both the cutoff frequency and surface roughness increase as the particle mass content increases. Their rates of increase change at 86 to 88 wt% for the PD-adsorbed PSP and at 92 to 94 wt% for the D-adsorbed PSP. These mass contents correspond to the CPVC, and the CPVC for the PD-adsorbed PSP is smaller than that for the D-adsorbed PSP. Note that the cutoff frequencies of PSP containing fewer particles than the CPVC are less than 150 Hz, which is the lowest achievable frequency using the acoustic resonance tube.

**Figure 6:**
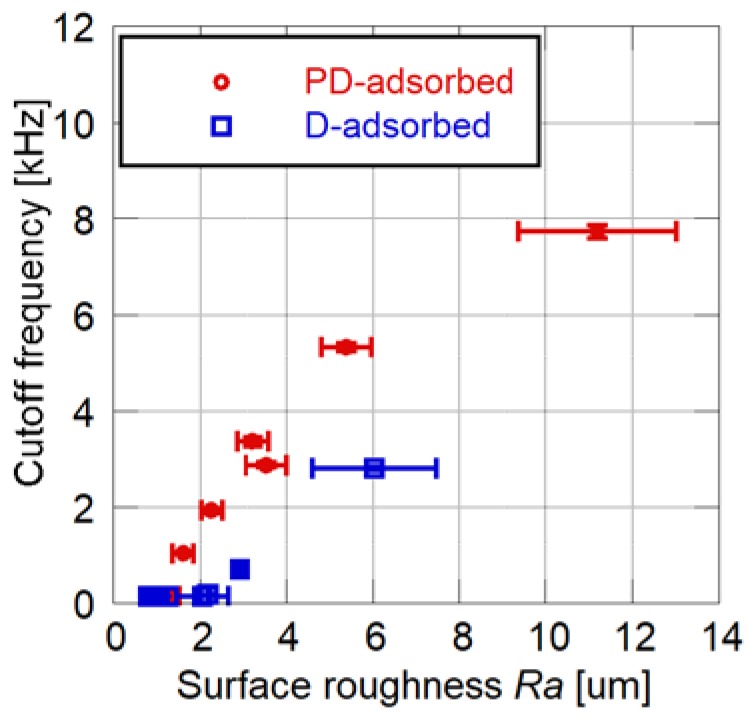
Relationship between cutoff frequency and surface roughness for the PD- and D-adsorbed PSPs.

The relationship between the cutoff frequency and the surface roughness is shown in Figure 6. For both the D- and PD-adsorbed PSPs, the cutoff frequency and surface roughness are strongly correlated. A PD-adsorbed coupon has a higher frequency response as compared to a D-adsorbed coupon, while maintaining the same surface roughness. The above results illustrate that the PD-adsorbed PSP outperforms the D-adsorbed PSP in terms of surface roughness and frequency response.

### 3.2. Effect of Solvent of Particle/Dye Solution

**Figure 7:**
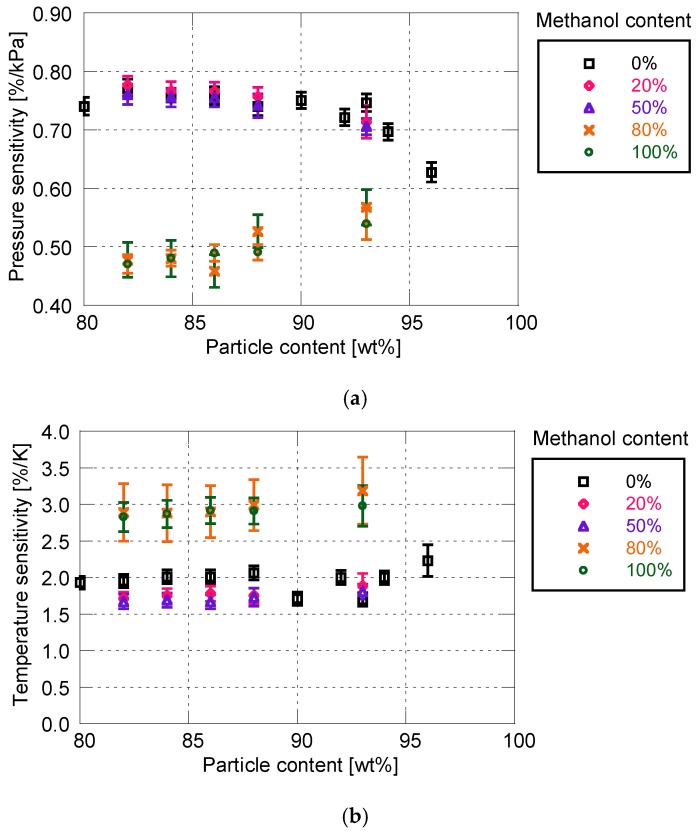
Static sensitivity characteristics related to methanol content for particle/dye solution. (**a**) Pressure sensitivity; (**b**) Temperature sensitivity.

Figure 7 shows the effect of solvent on the sensitivity characteristics for the PD-adsorbed PSP. As indicated in Section 2, the mixture of toluene and methanol is used as a particle/dye solution. The proportion of methanol to total amount is varied between 0 and 100%.

When the proportion of methanol is 50% or less, the methanol rarely affects the pressure and temperature sensitivities. However, at higher methanol proportions (i.e., 80 and 100%), the sensitivity of PSP is drastically reduced. The pressure sensitivity decreases by approximately 0.1 to 0.3%/kPa, and the temperature sensitivity increases by 1%/K.

**Figure 8:**
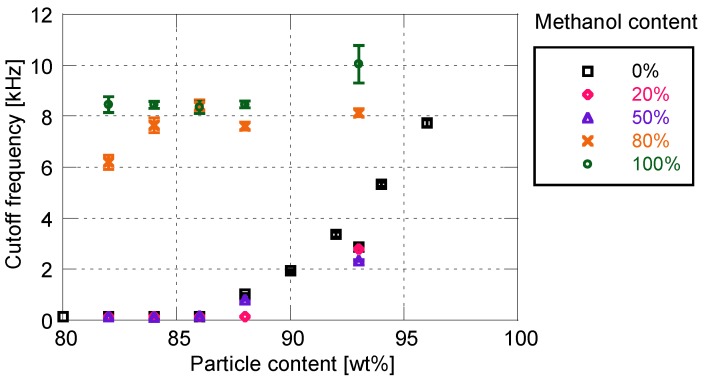
Cutoff frequency related to methanol content for particle/dye solution.

**Figure 9:**
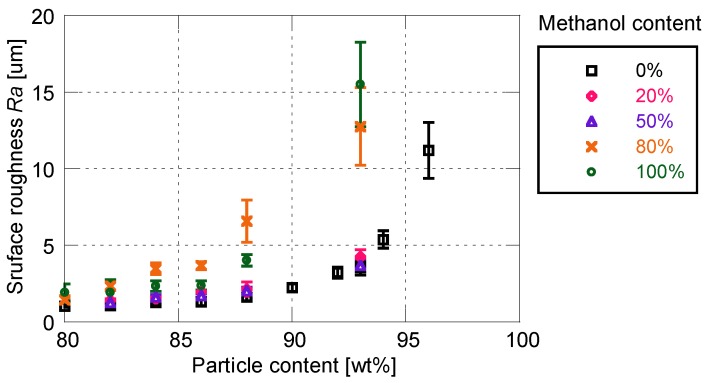
Surface roughness related to methanol content for particle/dye solution.

Figure 8 and Figure 9 show the effects of solvent on the cutoff frequency and surface roughness, respectively. Similarly to the sensitivity characteristics, the time response and surface roughness are not affected by the addition of 50 wt% or less of methanol. In this case, the cutoff frequency and surface roughness increase with the increase in particle mass content. When the proportion of methanol exceeds 80%, the time response is drastically improved to more than 6 kHz. The cutoff frequency changes little depending on the particle mass content, whereas the surface roughness changes significantly. When the ratio of methanol is greater than 80%, the increasing rate of surface roughness with respect to the particle mass content is much larger, as compared with the use of pure toluene as the particle/dye solvent.

**Figure 10:**
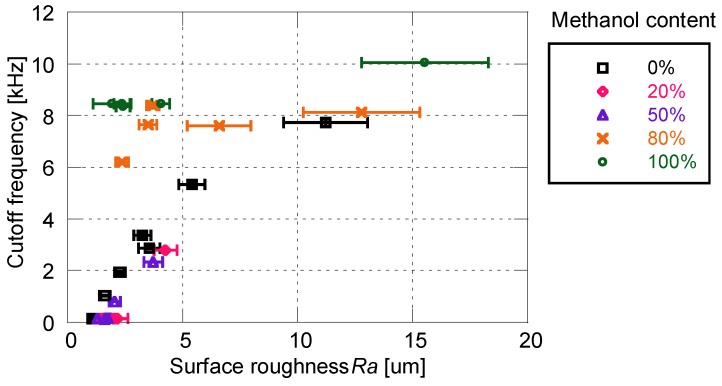
Relationship between cutoff frequency and surface roughness for the PD-adsorbed PSP with mixed solvent for particle/dye solution.

Figure 10 shows the relationship between cutoff frequency and surface roughness for the PD-adsorbed PSP with the mixed solvent. When the proportion of methanol is 50% or less, cutoff frequency correlates well with surface roughness. On the other hand, the correlation between the cutoff frequency and the surface roughness is eliminated when the proportion of methanol is 80% or more. The cutoff frequencies of the PSP are always higher than 6 kHz, but the surface roughness for these frequencies is scattered from 2 to 16 μm.

### 3.3. Microstructure

**Figure 11:**
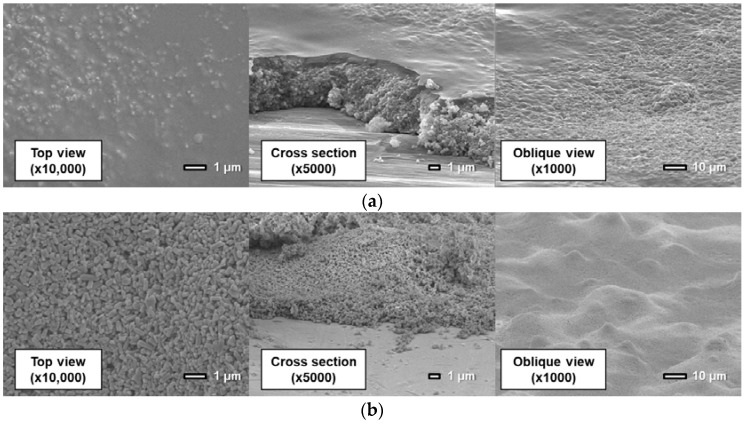
Scanning electron microscope images of the D-adsorbed PSP with a particle content of (**a**) 80 wt% and (**b**) 93 wt%.

**Figure 12:**
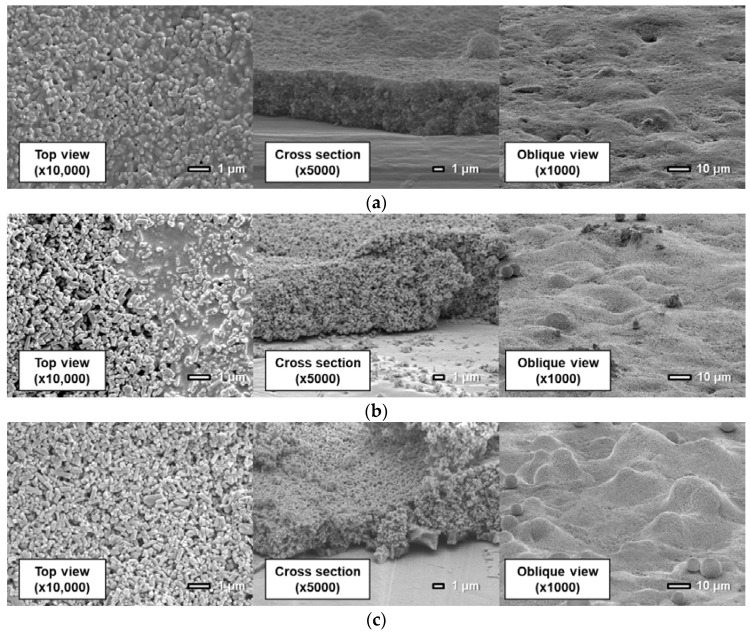
Scanning electron microscope images of the PD-adsorbed PSP the solvent of which is toluene with a particle content of (**a**) 80 wt%, (**b**) 90 wt%, and (**c**) 93 wt%.

**Figure 13:**
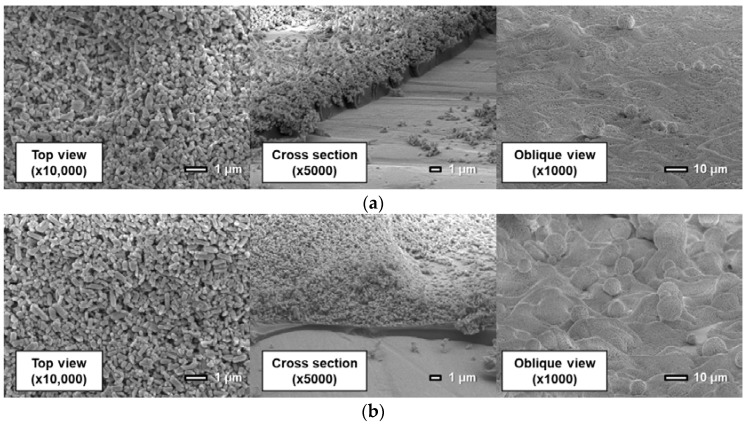
Scanning electron microscope images of the PD-adsorbed PSP the particle/dye solvent of which is the mixture (toluene: methanol = 0.2: 0.8) with a particle content of (**a**) 80 wt% and (**b**) 93 wt%.

Figure 11, Figure 12 and Figure 13 show surfaces and cross sections of PSP coupons imaged using an SEM. For the D-adsorbed PSP, images of coupons with particle mass contents of 80 wt% and 93 wt% are shown here as typical coupons. For the PD-adsorbed PSP, coupons with particle mass contents of 80, 90, and 93 wt% are selected for SEM observation in accordance with the acquired characteristics. The cross-section images are obtained by imaging the boundary between the coated region and the masked region.

Here, SEM images indicate that the coating film structure differs remarkably depending on the particle mass content and coating procedure. The following describes the characteristics of the coating film for each PSP coupon.

First, the coating film structure of the D-adsorbed PSP is investigated. When the coating film contains 80 wt% particles (Figure 11a), the coating film consists of two layers: a lower particle-rich layer and an upper polymer-rich layer. This state results from the high specific gravity of TiO_2_ particles. Before the solvent is volatilized and the film is formed, TiO_2_ particles sink to the lower layer by their own weight. When the coating film contains 93 wt% particles (Figure 11b), particles form small pores in the coating film. Moreover, polymer cannot be seen clearly in the image.

Figure 12 shows that the actual structure of the PD-adsorbed PSP differs from that predicted in a previous study (Figure 2b) when pure toluene is used for both the polymer solution and the particle/dye solution. Although particles and polymer are coated separately, particles and polymer are homogeneously distributed in the film. The pre-coated polymer film is dissolved again by toluene, which is used for the particles/dye solution. TiO_2_ particles are then dispersed into the dissolved polymer film. 

The coating film structure of the PD-adsorbed PSP with toluene solvent is varied depending on particle mass content. In Figure 12a for a coating film with 80 wt% particles, both polymer and particles can be observed in the top view. Figure 12b shows that the film surface is non-uniform and is divided into polymer-rich and particle-rich regions for the coating film with a particle content of 90 wt%. In the particle-rich region, pores with various diameters are formed. Since the coating film structure is unstable in the case in which the particle content approaches the CPVC, the local difference in film structure is easily caused by the difference in the volatilization rate of the paint droplets. When the coating film contains 93 wt% TiO_2_ particles, no polymer-rich region exists. This causes an improvement in the frequency response. In addition, large TiO_2_ particle aggregates are formed on the surface, and observed in the oblique view. This leads a rapid increase in surface roughness. The addition of a large amount of particles leads to the formation of huge particle aggregates as well as pores in the present polymer/particle system.

The use of a mixture of toluene and methanol as a solvent for the particle/dye solution causes a remarkable change in a coating film. Here, the proportion of methanol is 80%. Figure 13 indicates that the structure of the coating film is only slightly affected by the particle mass content. The coating film consists of two layers: the polymer layer and the particle layer. In other words, particles exist only in the outmost layer.

## 4. Discussion

The relationship between the cutoff frequency and the statistics of the brightness histogram of SEM images can provide useful insight into the mechanism of characteristic change and the design of faster PC-PSPs. In the present study, we focus on the skewness and kurtosis of histograms because the relative signal intensity in an image should be evaluated by SEM observation. The skewness and kurtosis were calculated in accordance with [16].

**Figure 14:**
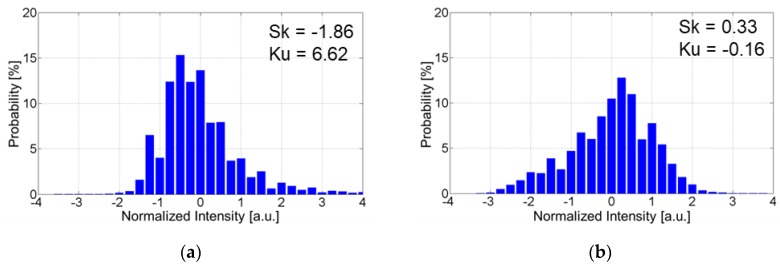
Histogram of SEM image for D-adsorbed PSP. (**a**) Particle content of 80 wt%; (**b**) Particle content of 93 wt%.

Figure 14 shows histograms of SEM images for the D-adsorbed PSP with particle contents of 80 wt% and 93 wt%. Histograms were obtained from top view SEM images (×10,000). In SEM images, white (bright), gray, and black (dark) areas correspond to particles, polymer, and pores, respectively. Skewness measures the degree of asymmetry of a distribution around its mean. 

Figure 14a indicates that the histogram is skewed left and skewness is negative for the D-adsorbed PSP with a particle content of 80 wt%. This means that a large surface area is occupied by polymer. On the other hand, the histogram is skewed right and skewness is positive for the D-adsorbed PSP with a particle content of 93 wt%, as shown in Figure 14b. This indicates the presence of many particles on the surface. Kurtosis measures the degree to which a distribution is more or less peaked than a normal distribution and indicates the homogeneity of coating surface. For the D-adsorbed PSP with a particle content of 80 wt%, the film surface is mostly occupied by the polymer. In this case, the histogram is relatively peaked and kurtosis is positive. For the D-adsorbed PSP with a particle content of 90 wt%, particles and pores appear on the entire surface. In this case, the histogram is relatively flat and kurtosis is negative.

**Figure 15:**
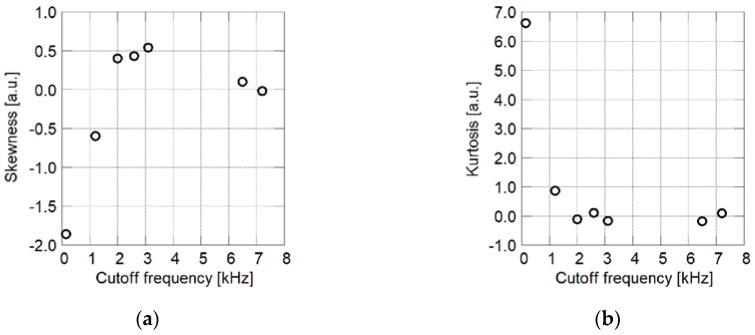
Statistics of brightness histograms of SEM images related to cutoff frequency. (**a**) Skewness; (**b**) Kurtosis.

In Figure 15, skewness and kurtosis are plotted with respect to the cutoff frequency. In the region in which the cutoff frequency is less than 3 kHz, skewness decreases with the increase in the cutoff frequency. This means that faster time response can be obtained when more particles cover the surface of the coating film. A PSP having a cutoff frequency of 6 to 7 kHz has higher skewness compared to PSPs having a cutoff frequency of 3 kHz. This corresponds to the formation of pores, which are observed as black regions in the SEM images. When the particle mass content exceeds a threshold, the film surface is covered primarily with particles. In this case, more pores are formed as the particle content increases.

For PSP exhibiting a cutoff frequency of less than 2 kHz, kurtosis decreases with the increase in the cutoff frequency. In this case, kurtosis is approximately zero and the corresponding cutoff frequency is more than 2 kHz. The decrease in kurtosis means the appearance of particles on the film surface. Once the film surface has been covered primarily by particles, kurtosis changes only slightly with particle mass content.

Based on the above considerations, the appearance of particles and the disappearance of polymer from the film surface are concluded to correspond to the increase in cutoff frequency. In other words, the time response of a PC-PSP is governed primarily by the surface structure on the micro-scale, which is shown in top view SEM images (×10,000).

**Figure 16:**
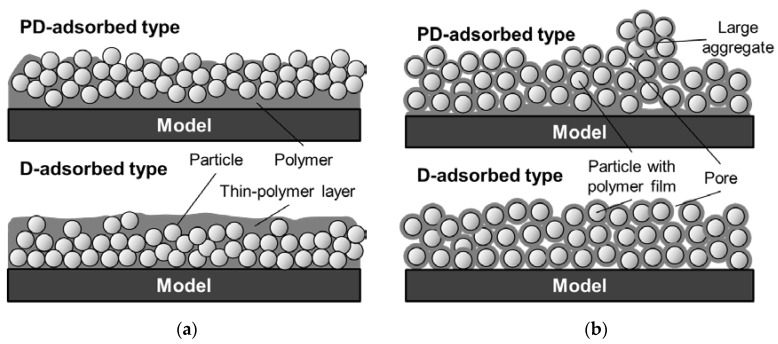
Modified sketches of structures of PC-PSP with toluene solvent (**a**) when the particle content is less than the CPVC and (**b**) when the particle content exceeds the CPVC.

Based on the SEM images, the film structure can be modified as shown in Figure 16. When the particle content is smaller than the CPVC (Figure 16a), the vertical distribution of particles is affected by the coating procedure. Particles and polymer are mixed in the coating film for the PD-adsorbed PSP, but more particles are distributed near the surface, as compared to the D-adsorbed PSP. This leads to the formation of pores on the surface and improves the time response. When the particle content exceeds the CPVC (Figure 16b), the film structures of the PD- and D-adsorbed PSPs are similar. SEM images suggest that particles are covered with a thin-polymer film and are uniformly distributed in the coating film. Large aggregates are easily formed for the PD-adsorbed PSP because the amount of polymer is small near the surface. In summary, the coating procedure does not cause a significant change in film structure but results in a change in the distribution of particles and polymer in a coating film. In addition, the time response of a PC-PSP is significantly affected by the amount of particles and polymer near the film surface.

It is necessary to discuss why the use of methanol affects PC-PSP characteristics and why the rapid characteristic change occurs between 50% and 80% methanol content. First, the difference in solubility of the polymer with respect to toluene and methanol should be considered. The solubility of the polymer with respect to toluene is high because toluene is a non-polar solvent. Polymer film, which is the first layer of the PD-adsorbed PSP, is redissolved by toluene, and dye and particles then penetrate the film. On the other hand, the polymer film is not dissolved with methanol because the solubility of the polymer with respect to methanol, which is a polar solvent, is low. Consequently, luminophore is not distributed in polymer film, but rather on the particle surface. This causes the improvement in the time response. Second, the rapid characteristic change occurs between 50% and 80% methanol content may be the azeotropic behavior of the mixture. A mixture of 29% toluene and 71% methanol (by mass) is an azeotrope. The compounding ratio of the azeotrope corresponds well with the threshold at which the change in the characteristics occurs.

## 5. Conclusions

We improved the characteristics of PC-PSP by using a new coating procedure and compared new and conventional procedures. The effect of particle mass content on PSP characteristics was investigated for both of D- and PD-adsorbed PSPs. In addition, the effect of solvent was evaluated for the PD-adsorbed PSP.

As a result, the difference in the characteristics between the two types of PSPs was clarified. Although the surface roughness and time response increase as the mass content of particles increases for both the D- and PD-adsorbed PSPs, the CPVC for the PD-adsorbed PSP is smaller than that of the D-adsorbed PSP (88 wt% and 93 wt%, respectively). The PD-adsorbed PSP has a higher frequency response as compared with the D-adsorbed PSP, while maintaining the same surface roughness. Consequently, the PD-adsorbed PSP outperforms the D-adsorbed PSP in terms of surface roughness and frequency response.

SEM observation showed that the increase in time response of the PC-PSP is explained primarily by the micro-scale surface structure. The presence of particles and pores on the film surface is concluded to correspond to the increase in cutoff frequency.

Although particles and dye are adsorbed onto the polymer film, particles and polymer are uniformly distributed in the polymer film. More particles are distributed near the surface for the PD-adsorbed PSP, as compared to the D-adsorbed PSP. This leads to the formation of more pores on the surface. Hence, the frequency response of the PD-adsorbed PSP is faster than that of the D-adsorbed PSP containing the same amount of particles.

## Figures and Tables

**Table 1:** Preparation conditions of PC-PSPs.

Type	Solvent for Dye		Particle Mass Content [wt%]										Mix Ratio (polymer:toluene)
D-adsorbed	Toluene		80	82	84	86	88	90	92	93	94	96	0.25 g: 15 ml
PD-adsorbed	Toluene		80	82	84	86	88	90	92	93	94	96	0.25 g: 10 ml
	Mixture			82	84	86	88			93			
